# Exploring service users’ and healthcare professionals’ experience of digital and face-to-face Health Checks in England: a qualitative study

**DOI:** 10.1136/bmjopen-2024-090492

**Published:** 2025-03-13

**Authors:** Chloe Forte, Elisabeth B Grey, Patricia Jessiman, Hugh McLeod, Ruth Salway, Carlos Sillero-Rejon, Rebecca Harkes, Paul Stokes, Frank De Vocht, Rona Campbell, Russell Jago

**Affiliations:** 1 Population Health Sciences, University of Bristol, Bristol, UK; 2 NIHR ARC West, Bristol, UK; 3 London Borough of Southwark, Public Health, London, UK

**Keywords:** QUALITATIVE RESEARCH, eHealth, Health Services Accessibility, Quality in healthcare

## Abstract

**Abstract:**

**Introduction:**

In England, eligible adults aged 40–74 years are invited to attend a face-to-face (F2F) NHS Health Check appointment every 5 years. A digital version of the Health Check was introduced by a local authority as an alternative for those hesitant or less able to attend an F2F appointment.

**Objectives:**

This qualitative study aimed to understand service users’ (SUs) and healthcare professionals’ (HCPs) experiences and opinions of F2F Health Checks and digital Health Checks (DHC), identify barriers and facilitators of the F2F Health Check and DHC pathways, and recommend potential improvements.

**Design:**

This is a qualitative study, involving interviews with a purposive sample of participants.

**Participants and setting:**

A purposive sample of 30 SUs and 8 HCPs were recruited by an external market service company in the London Borough of Southwark.

**Methods:**

Semistructured interviews were conducted, which included questions on understanding why SUs chose a type of Health Check, their experiences of the service and suggestions for improvement. HCP interviews covered HCP experiences of providing both services, including any impact on workload. The Framework method of thematic analysis was used to analyse the data.

**Results:**

SUs identified benefits of the DHC service including its convenience, ease of use and access. Both SUs and HCPs acknowledged the limitations of the DHC, including self-reporting physical measures (eg, blood pressure and cholesterol levels) or difficulties going elsewhere to measure them, and the lack of opportunity to discuss health with a professional. SUs and HCPs both noted the lack of available appointments and time constraints as barriers associated with the F2F service.

**Conclusions:**

Both HCPs and SUs perceive that in its current form, the DHC has benefits and barriers to its use. If these are adequately addressed, the DHC may help address the demand and pressure within General Practitioner (GP) clinics.

**Trial registration number:**

This study was registered on the Open Science Framework: https://osf.io/y87zt/.

**STRENGTHS AND LIMITATIONS OF THIS STUDY**

- A strength of the study is the focus on service users’ (SUs) real experience of the digital Health Check (DHC) and the face-to-face Health Check.
- Semistructured qualitative interviews used a topic guide to ensure that data collection was rigorous and robust.
- Data were collected in the London Borough of Southwark that was examining the use of DHC as a form of innovation in NHS Health Check provision.
- A limitation of this study was that only one healthcare professional was aware of and had experience with the DHC service.
- The majority of SUs interviewed were of white ethnicity, which limits the generalisability of the findings.

## Introduction

 Integrating digital technology into healthcare is key to improving efficiency and equity of access.^1^ Since 2015, the UK National Health Service (NHS) has prioritised digital-first primary care,^2^ enabling remote consultations and symptom checks.^3^ The 2019 NHS Long Term Plan aimed to provide digital access to primary care by 2023/2024,^4^ with GP practices mandated to offer online consultations by 2021.^5^

The integration of digital technologies in healthcare is not new, but their efficacy alongside standard care remains under-researched. A 2019 evidence review for NHS England highlighted the benefits of offering alternatives to in-person care, such as greater convenience, improved access for those with mobility issues and reduced stigma through increased privacy.^6^ Since the COVID-19 pandemic, online tools have become widely adopted for tasks like prescription requests, scheduling and consultations.^7^ With rising demand for appointments and a shortage of healthcare professionals (HCPs),^8^ there is a clear need for a hybrid model combining digital and face-to-face (F2F) care. It is estimated that 40% of appointments at a GP clinic could be either transferred to other locations or completed by an HCP who is not an GP.^9^ Using a model of care that uses both digital and standard care approaches may allow for greater flexibility^10^ and quicker navigation through the care system as well as alleviate pressure in GP practices.

The 2019 NHS review found that digital and remote care is primarily used by younger, female, individuals with English as their first language, higher incomes and education levels,^6^ raising concerns about increased health inequalities for older adults and disadvantaged groups. Clinicians worry that remote delivery may miss important cues and symptoms, leading to more ‘safety-netting’ practices like unnecessary antibiotic prescribing.^6^ While digital tools may ease pressure on primary care, improve flexibility and offer economic benefits,^6^ their impact on staff workload is unclear. Barriers include poor infrastructure and lack of staff training,^6 11 12^ which are essential for accuracy, confidence and adoption. Understanding benefits and challenges from both patient and staff perspectives is vital for effective implementation.

This paper reports the evaluation of a digital version of the NHS Health Check, developed by Southwark Council, a local authority in the southeast of England. The NHS Health Check programme, commissioned by local authorities, aims to identify and manage early signs of cardiovascular disease, type 2 diabetes, kidney disease, stroke and dementia every 5 years for adults aged 40–74 years.^13^ Traditionally conducted F2F in GP practices, the Health Check assesses seven key risk factors of noncommunicable diseases: physical inactivity, excess weight, smoking, alcohol consumption, high blood pressure, high cholesterol and impaired glucose processing. It offers follow-up clinical assessments and behavioural support to reduce disease risk and address health inequalities. Despite mixed reviews on the overall effectiveness of health checks, evidence suggests that they improve the detection of risk factors.^13^ Health Checks are standardised to ensure the quality and safety of the programme,^14^ but local authorities do have some flexibility over how they are delivered, for example, prioritising invitations to ‘high risk’ individuals.

**Figure 1:**
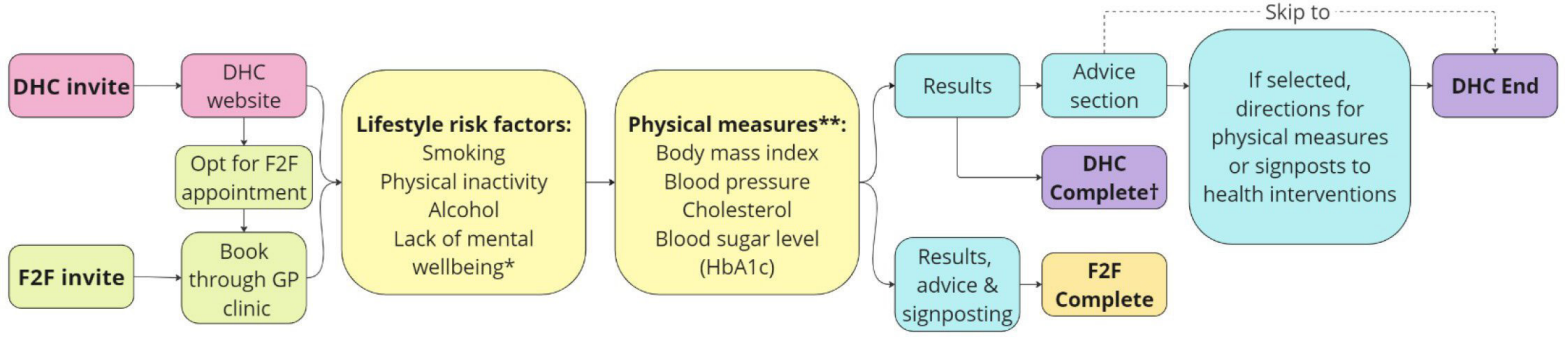
Flow diagram showing an overview of F2F Health Check and DHC pathways. *Only included in DHC. **For DHC if values of physical measures are known from the last 6 months they are inputted here, if not a population average is used. For F2F, physical measures can be taken either during an appointment or at a follow-up appointment. †A complete DHC is interpreted as reaching the results page with or without physical measures. DHC, digital Health Check; F2F, face to face.

The digital version of the NHS Health Check (DHC) replicates the F2F process as an online survey where users answer health- and behaviour-related questions. On completion, they receive immediate feedback, with results sent to their GP. If concerning risk factors are identified, users are advised to schedule an F2F appointment. Users can also select health priorities and receive personalised advice, such as measuring physical measures (eg, getting their blood pressure, cholesterol or blood sugar measured at either a pharmacy, GP clinic, leisure centre or an at-home blood test) or accessing support for adopting healthier behaviours. The DHC process may present a more acceptable alternative that still enables the delivery of preventive advice and the identification of early-stage disease. For a full breakdown of the DHC service, see Salway *et al*.^15^ See figure 1 for a flow diagram of the DHC and the F2F Health Checks.

This qualitative study aimed to understand service users’ (SUs) and HCPs’ experiences and opinions of F2F Health Checks and DHCs, identify barriers and facilitators of the F2F and DHC pathways, and recommend potential improvements.

## Methods

### Study design

A qualitative design was adopted for this research using one-to-one semistructured interviews with a purposive sample of SUs (individuals who had been invited to either F2F Health Check or DHC) and relevant HCPs from the target area identified by GP practice managers. This qualitative study was part of a wider evaluation study to compare the uptake of NHS Health Checks between those invited to the DHC and those invited to the F2F Health Check. Detailed methods for the evaluation^16^ and a full-service evaluation of the uptake and effectiveness of the DHC pathway are reported elsewhere.^15^ Original project protocol is presented in the Supplementary materials section (Online supplemental file 2). This project has received ethical approval from the East Midlands (Nottingham 1) NHS Research Ethics Committee (ref: 22/EM/0280). The Standards for Reporting Qualitative Research were used to guide reporting.^17^ Online supplemental table 1 presents the checklist.

### Participant recruitment

The setting for the evaluation study was the London Borough of Southwark in England, where invitations to complete a Health Check were sent out to 9000 eligible SUs randomly selected from theEgton Medical Information Systems or EMIS, the electronic patient health record system in North Southwark. SUs were invited to either the F2F Health Check (n=3000) or the DHC (n=6000). SUs who received an invite to the DHC were able to book an F2F Health Check at their GP practice if they preferred, whereas SUs who received the F2F Health Check invite were not given the option to complete the DHC. Overall, 20% of patients completed a Health Check. Of those assigned to DHC, 21% completed the DHC Health Check, and a further 3% chose to complete an F2F Health Check, compared with 11% of those assigned to F2F who completed an F2F Health Check. Those who completed any type of Health Check included higher proportions of women, those with a family history of cardiovascular disease (CVD) and those from less deprived areas. Those who completed an DHC compared with an F2F Health Check included more men, those from white ethnicity, those with low diabetes risk and fewer with overweight or obesity. A full breakdown of demographic information of those involved in the service evaluation is reported elsewhere.^15^

For the current qualitative study, the SU participant group was recruited using the market research company, Leftfield. An invitation was sent by an external company (iPlato) via SMS to all SUs who were invited to a Health Check (both digital and F2F) between January and March 2023. Leftfield screened responding SUs to recruit a sample of participants to represent a range across the following criteria: Health Check completion status (ie, completion of F2F Health Check, the DHC or did not complete a Health Check), gender, age, ethnic groups and area of residence. Selected participants were sent an electronic consent form. When consent had been given, telephone/online interviews between participants and a researcher were organised.

All GP practices in the target area were sent invitations for HCPs to take part in an interview. Invitations were sent on behalf of the research team by the GP Federation to GP practice managers, who were asked to forward the invitation to relevant HCPs. The invitation directed the HCP to an online form where a full participant information sheet was available to read and download before completing a consent form, a demographic survey and a contact detail form. The research team then contacted the HCP to arrange a suitable time for an interview. The study aimed to recruit 30 SUs and 10 HCPs.

### Procedure

All interviews took place via telephone or video call, according to participant preference, and were conducted by experienced qualitative researchers (EBG and PJ). The interviews were semistructured, which allowed the researcher to adapt the questioning according to the participant’s earlier responses and prompt for further information if relevant novel issues were raised.^18^ Participants completed an online consent form prior to the interview, but the researcher checked their understanding of the interview procedure and how their data would be used at the start of their meeting. Interviews lasted approximately 30 min and were audio recorded using an encrypted digital recorder and then fully transcribed verbatim. Participants were offered a £50 Love2Shop gift voucher for taking part.

### Materials

Interview schedules for SU and HCP interviews were co-developed by the whole project team, with input from the Public and Patient Involvement (PPI) group (see the Patient and public involvement section). Interview schedules were created based on the intervention logic model and study research aims. Briefly, SU interviews sought to understand why SUs chose either an F2F Health Check, DHC or neither, their experience of the service and in what ways the service could be improved. Interviews with HCPs covered their experience of providing the combined Health Check service, including any impact on workload for them and their colleagues, any concerns or perceived benefits of the service and any suggestions for improvements. The research team revised the schedules based on the progress of early interviews (eg, including more information about the DHC for HCPs who were not aware of them). The final interview schedules for both SUs and HCPs are presented in the Supplementary materials section (online supplemental files 3 and 4).

### Patient and public involvement

When designing this research, we consulted a PPI group, recruited from the local borough’s Healthwatch network and comprising eight residents aged between 40 and 59 years. The majority of the group was female and of black ethnicity. Through an online meeting, the group provided feedback and suggestions on the proposed protocol and research materials. Two members of the group joined the project steering committee to provide ongoing advice and oversight from an SU’s perspective.

### Analysis

Interview transcripts were analysed using the Framework method of thematic analysis.^18 19^ Separate analyses were conducted for SU and HCP interviews. After reading all transcripts, draft analytical frameworks for HCPs and SUs were developed by CF and TJ including themes and subthemes that were driven by the data but were also relevant to the research objectives. In the Framework method, a qualitative code book is referred to as an analytical framework. This is created when the researchers have coded the first few transcripts independently and then meet to compare labels and agree on a set of codes to apply to all subsequent transcripts. These codes can be grouped into categories and are clearly defined.^19^ The draft analytical frameworks were used to code a subsample of the transcripts by CF and TJ, and then they were reviewed and amended as necessary to ensure that the frameworks captured all the pertinent information for this study. The analytical frameworks were entered in the NVivo software^20^ and applied to all transcripts. Analysis was an iterative process—the team regularly reviewed and revised the frameworks to ensure that they remained a good ‘fit’ for the data. The final analytical frameworks are available in the Supplementary materials (online supplemental files 5 and 6). When all transcripts were coded, a framework matrix was developed with columns to represent each subtheme and rows for each participant. Cells were populated with quotations, data summaries and the researcher’s analytical notes. This ‘charting’ method created an accessible dataset through which themes and subthemes could be explored by respondent type. A summary of the data under each subtheme was developed to inform the next stage of the analysis, moving up the analytical hierarchy to explore patterns and associations between themes in the data.^21 22^

## Results

A total of 30 SUs and 8 HCPs completed semistructured interviews. Table 1 describes the sample characteristics. A summary of the main themes and subthemes for both SU and HCP interviews is described in table 2.

**Table 1:** Sample characteristics

	SU samplen=30	HCP samplen=8
Gender*		
Male	12	1
Female	17	7
Age group (years)		
Under 60	9	8
60+	21	0
Ethnicity		
White ethnicity	21	4
Other ethnicity (black African, black other, Asian, other)	9	4
Health Check type attended (SUs)		
Completed DHC	15	
Completed F2F	11	
Unsure	1	
Completed none	5	
DHC experience (HCPs)		1

* Excluding other, refused or not reported.

DCHdigital Health ChecksF2Fface to faceHCPhealthcare professionalSUservice user

**Table 2:** Summary of themes and subthemes for SU and HCP interviews

Themes	Subthemes SUs	Subthemes HCPs
F2F barriers	Challenging booking processLack of available appointments	Time constraints during appointments
F2F benefits	Ability to discuss health in person	Ability to discuss health in person
DHC barriers	Lack of health discussionsCannot add individual context to lifestyle question responsesPhysical measures (mixed responses)Cannot discuss resultsDHC advice too general	Lack of health discussionsAccuracy of responses and measuresDHC follow-up
DHC benefits	Easy-to-use websiteConvenienceAvoid GP clinicHealth ownershipRemote benefits (privacy, etc)	Awareness of health checksConvenience

DHCdigital Health CheckF2Fface to faceHCPhealthcare professionalSUservice user

The findings are presented according to the benefits and barriers of both types of Health Checks (DHC and F2F). Anonymised quotations are included for SUs and HCPs. SUs’ Health Check status is described (F2F, DHC, both or none) and whether they have experience with DHCs for HCPs.

### Benefits of F2F Health Check

The majority of the benefits identified by both HCPs and SUs for the F2F Health Check were items that were identified as barriers for the DHC, described below, including being able to discuss health with a trained professional, adding context and individual factors to questionnaire responses, receiving immediate feedback and answers and scheduling follow-ups immediately if necessary.

### Barriers to F2F Health Check

The most prominent barrier to the completion of F2F Health Checks for SUs was the difficulty in making an appointment and long waiting times in busy GP practices.

What bothered me is going to the GP physically, queuing there for I don’t know how long. Then, even if you have a slot where you should be, they always overflow time wise. And my issue is I don’t have time. With three kids, working full time, I don’t have… Sorry, I can’t spare a minute left or right. (SU12, DHC)

HCPs also expressed that the lack of available appointments was a major barrier to F2F Health Checks. In addition to this, HCPs perceived that the time they have allocated for an F2F Health Check (according to interview findings, some HCPs noted around 15–30 min, depending on the practice) is sometimes not enough to complete the lifestyle questions (ie, smoking, physical activity and risk factors) and the physical measures (height, weight, cholesterol, blood pressure and diabetes check), and then go through the results and the follow-up advice with patients. HCPs report that Health Checks vary by person, and for some patients they need to go over the allocated time in order to adequately perform the service. They will also email follow-up advice and services to patients when they do not have the information at hand.

I have to say that we’re getting less time to do them. So, it has to be quite short in terms of- So, say if somebody came with a list of issues, you would have to signpost them and deal with that. But you can’t- Unfortunately, I feel like in the old days, I think we had half an hour. Then they cut it to 20 min. (HCP04, No DHC experience)

### Benefits of the DHC

SUs identified many benefits of the DHC. Notably, those who completed an DHC were able to identify more benefits of the service than those who had not. However, those who completed an F2F Health Check felt that the inclusion of an online option would improve the service.

One of the main benefits mentioned was that the DHC was straightforward and easy to use. SUs noted that it matched the presentation of other NHS online surveys and forms which was helpful as it was recognisable. SUs noted that all questions in the survey were easy to understand.

The DHC was also convenient, as SUs did not need to arrange an appointment with their GP practice. Most identified this as a clear benefit. It could be completed any time of day, and SUs could take their time going through it. It was also noted that the text message link was easy to access for SUs.

It’s convenience online, at least I can do it from the comfort of my home. (SU05, DHC)

Some SUs mentioned that we are in a *digital age* and that the DHC adapts to that and gives people more options. The risk of contracting COVID-19 meant some SUs’ view not having to attend the GP clinic as a key benefit of the DHC service. Additionally, as GP clinics are currently experiencing severe pressure to accommodate appointments, having the option of doing things online removed SUs from experiencing the frustration of making an appointment and partly alleviated the pressures within GP clinics and the NHS. Furthermore, as SUs were doing the survey independently, it led to them taking ownership of their health and understanding it more, giving SUs an active instead of passive role in this process.

…if I have to do something for myself then I’m actually more aware of what I’m doing and why I’m doing it rather than just go to the doctors and then forget about it. (SU26, F2F)

Other notable benefits reported by SUs included that the DHC was helpful for those who are introverted as they do not need to talk to other people about sensitive topics. The DHC was helpful if there is a language barrier as SUs can take their time with the survey and look up anything they are unsure of. Finally, some users noted that there is no perceived judgement with the DHC as there might be when completing the lifestyle questions with an HCP. SUs felt they were not ‘confessing’ anything.

The main benefits of the DHC identified by HCPs included that it was another way to raise awareness of Health Checks in general. When individuals receive an invite to the DHC, they can choose to do it online or they may choose the option to do it in the standard way in a GP clinic. Regardless, it increases awareness and provides another method of completing the Health Checks.

### Barriers to the DHC

There was a range of responses regarding barriers to the DHC from both SU and HCP perspectives. Half of the HCPs interviewed had only recently heard of the DHC; the remaining half were not aware of the DHC. One HCP had experience with a patient who had used the DHC and then returned to the clinic for follow-ups. Once the interviewer mentioned the DHC, most staff members were interested to know more about it and how the physical tests were measured. Half of the SUs interviewed had experience with the DHC.

A recurring theme in the data was that SUs could not communicate with a health professional immediately during the DHC. This was seen as a concern for a range of reasons, such as the inability to ask questions and discuss health issues, inability to request additional assessments, inability to add context to answers in the health assessment, difficulty in scheduling follow-up appointments and lack of opportunity for HCPs to detect other health issues such as mental health symptoms (for clarity, the F2F Health Check does not test for symptoms of ill mental health or provide additional tests; however, SUs have the potential to request additional tests or discuss health concerns during the F2F appointment, which is not possible during the DHC and would need to be addressed as an additional option following on from the digital service).

Well, it’s a completely different experience when you see a doctor in person than online. Online you just follow what they offer you, but in person you can ask questions. (SU07, F2F)

Similarly, many of the HCPs expressed concern at the lack of opportunity to assess the SUs themselves and give them positive feedback on lifestyle changes.

Yes, we still say, “So, this is good. It could be because you probably exercise a lot, or if someone is slim but admits to bad diet, can warn them that this might be precursor to high cholesterol. Would this happen online? (HCP01, No DHC experience)

Another barrier to care from the DHC was the forced response nature of the online survey. Not being able to justify answers was frustrating for some SUs. Similarly, SUs found getting their results online worrying as they do not have the opportunity to discuss their results with someone immediately in order to ensure understanding. Examples of results given from the DHC are displayed in the Supplementary materials section (online supplemental file 7).

I found it quite general and a bit anxiety-inducing, because it did come back with quite harsh results. It categorised me as someone who will have premature heart problems or likely to have heart problems or other issues that surprised me. Yes. I don’t think are justified with my general lifestyle. (SU28, Both)

So I’m 75, so if I've got the heart of an 85-year-old, does that mean I'm totally knackered already, I better watch out? I don't know what it meant. (SU18, DHC)

Some HCPs were unsure if the data entered by SUs into the DHC would be accurate, due to lack of understanding, human error or even potentially dishonest reports. HCPs have no way of verifying the information when it is completed remotely. Furthermore, HCPs were not confident in the accuracy of the physical measures if completed by SUs at home rather than by professionals.

You can kind of tell when somebody is not being wholly honest in an appointment. You can't tell that from someone inputting information. (HCP02, No DHC experience)

The physical measures were also perceived as a barrier for HCPs, and there were different attitudes towards them from the SUs’ perspectives. In the DHC, SUs are asked if they know their blood pressure, blood sugar, height, weight and cholesterol levels and then they are required to input the measurements. If they do not know their measurements, they can proceed, and their risk scores are calculated from national average values. This was seen as a barrier to completing the DHC survey as some SUs interpreted the initial question to mean they would not be able to finish the survey as they did not know their measurements, thus leading to early drop-off and failure to finish the survey. Most SUs did not see the physical assessments themselves (ie, doing the tests at home via a postal kit, at a leisure centre, pharmacy or GP clinic) as a barrier to the DHC (see quotes below). However, in this sample, only one SU chose to do the tests at home using a kit and postal service; they found it very difficult to complete as a high degree of dexterity was required. Other SUs were asked their opinion about using the kits and some said that it would not be an issue but perceived that it could be for others. One SU reported that they were directed to buy a device to measure blood pressure (possibly indicating that the web link they were sent to order a blood test kit misdirected them or that the user misunderstood the instructions) and mentioned going to a pharmacy to do the tests costs money, (potentially referring to travel costs as the actual test is free for the user), which was a barrier. Physical measures present an additional step that SUs need to take in order to fully complete a Health Check following the DHC survey, which would be completed as part of the F2F Health Check.

Oh wow, okay, that’s a new concept. I’ve never ever taken my own blood and taken it to the wherever. I’ve always gone to the hospital to have my blood done. I’ve never ever, oh my goodness. Alright, but I wouldn’t do that, I would not go, you know. You would have to send me to get my blood done. I’m not going to take my own blood. (Laughter) (SU01, None)

Because we can all do a blood pressure check, we could do a finger prick check, you know, it’s not exactly hard to do, do our weight and height, we could do that and send that through and put the stats on our own record. But I understand I would probably be more proactive with using the app and stuff like that. I mean I’m quite okay to be proactive in that way. (SU26, F2F)

HCPs were not convinced that users would fully engage with the DHC process as there are many stages where drop-out could occur (ie, waiting for blood kit, sending blood, waiting for results, then follow-up appointments), whereas everything is completed in one appointment in the F2F Health Check, or a follow-up scheduled at the initial appointment.

I feel like people would then just be put off from doing it but if they just know that they can have it all done in the one go, it’s just going to take 25 minutes of your time, rather than completing this survey, sending it off… It then takes a couple of weeks, you know. (HCP06, DHC experience)

HCPs also queried whether the DHC would save time, as SUs completing the DHC without up-to-date physical measurements, and who want to get these measured, would still be advised to attend the GP clinic/pharmacy/leisure centre. Additionally, those who are identified as ‘high risk’ would also be advised to schedule a follow-up appointment at a GP clinic. One HCP reported that a patient came in to get their blood taken after completing the DHC, but as a staff member did not understand the results from the DHC, they completed the Health Check again with the patient.

I feel like it’s a good idea, but it could be improved. I think I feel like more… Like I said, I don’t know what information is going into the digital Health Check because it’s not filtering down to me when they come back to see me for a blood test. No, I mean there’re things going onto there but I… You know, they end up with a Q risk, they end up with a Health Check thing, but there’s no breakdown of what’s been… I don’t actually know. They just come up to me and then I end up having to do a full Health Check, basically. (HCP06, DHC experience)

Several SUs had issues trying to recall the results of their DHC and were unsure where to locate them. Additionally, if users completed a home blood test (which was conducted by a third-party provider commissioned by the local authority), they received their results in an email directly from the provider, which also caused confusion with information received following the DHC. Similarly, if a user completed a physical measure through a separate provider, users were worried that the results would not be communicated back to their GP or uploaded to their medical records. SUs who completed the DHC also struggled with the ‘medical jargon’ included in the report. Many users commented on being unsure how to interpret the results. In contrast, users who attended the F2F Health Check were able to recall and interpret their results. Not being able to take the SU through their results to ensure that they understand and know the follow-up steps and what is available to them was a disadvantage of the DHC from the HCPs’ perspective.

Other barriers to the DHC included the behavioural advice given following DHC completion. Many users found that the advice was not individualised enough to their personal situation. As an example, the DHC did not give advice on financial help for healthy living to users struggling financially. It must be noted that the DHC asks users to highlight perceived barriers to healthy behaviours to give them personalised advice based on this. For example, in terms of financial and access barriers to healthy eating, there are two options that individuals can highlight: ‘I cannot afford to eat well’ and ‘I do not have access to healthy food’. If these are selected, then the individual will be signposted to advice tailored to these barriers (including NHS Eat Well for Less schemes and food access services in the area). Additionally, the advice given at an F2F appointment is similar to the DHC, and the difference is it is typically delivered by an HCP with an opportunity for discussion with the user. Regardless, users still felt the advice given was too general in the DHC.

## Discussion

To the best of our knowledge, this is the first qualitative study exploring HCP and SU experiences and opinions of DHC and F2F Health Checks. This study found similar benefits and barriers to using digital services in more general primary care,^6^ such as convenience and ease of use of the Health Checks as benefits, and the lack of human contact as a perceived barrier. SUs also noted key barriers to the F2F Health Checks, mainly stemming from a lack of available appointments, and HCPs noted pressure with completing the Health Check during the allocated time. The DHC may present a potential supplementary option to the standard Health Check system in this area.

A concern identified throughout the interviews was that the NHS is under pressure, evidenced by patients experiencing long waiting times and staff not having adequate time or resources available to conduct the Health Checks appropriately. In the United Kingdom, GPs are experiencing unsustainable workloads.^23^ Also adding to the pressures on GP clinics is the lack of adequate staff and resources allocated to the service as the population grows, and increases in patient consultations as people are living longer with complex health needs.^24^ These issues present a considerable source of challenge for all and frustration for both HCPs and patients. The majority of participants interviewed acknowledged these issues and expressed a desire to help alleviate the pressure. Even participants who were unaware of the DHC suggested that the inclusion of an online option to attempt to target these waiting times at GP clinics could be a potential solution. This suggests that both SUs and HCPs may be open to the DHC, which may aid with the implementation of the service.

There were a range of benefits noted for the DHC service. Participants stated that one of the prominent benefits is convenience. It can be completed at any time and it does not need to be completed in one sitting. This is a direct contrast to the long and frustrating experiences patients and staff alike noted while trying to secure an appointment at a GP clinic. Additionally, SUs mentioned that the DHC survey was straightforward and easy to navigate. No users mentioned any issues in understanding the lifestyle questions making it a viable option to complete the lifestyle questions without the help of an HCP thus relieving pressure on the NHS system.

In its current design, issues arose throughout the DHC particularly with the physical measures, as SUs identified these as the first roadblock of the service. If SUs do not have their results at hand, they need to organise measurements themselves and return to update their results. The service prompts users to do this; if they select it as a priority, it provides links to book the tests and links that direct them to the page where they can update their results. This begins the patient-driven nature of the DHC that is distinct from the F2F Health Check’s more passive approach. Additionally, HCPs identified that there are many steps to completing the DHC beyond simply clicking the link and completing a survey. SUs need to initiate every step and read a report of their results online, whereas with the F2F Health Check usually patients are led through the appointment by the HCP and have their results and follow-up advice explained, if time permits. SUs need to be motivated to properly engage with the DHC, their results and their suggested follow-ups. Motivation and attitudes have been highlighted in previous studies as important factors for benefiting digital services.^25^,^27^ This suggests that potentially the DHC is suitable for health-conscious, motivated individuals and could be offered alongside F2F Health Checks as an alternative model that suits individuals more. Additionally, DHCs could be targeted to those who potentially would not attend an F2F appointment due to barriers in F2F (eg, time constraints, introversion, perceived judgement and language barriers) and in turn increase Health Check uptake.

One of the issues identified with the DHC was the lack of human contact with an HCP. Conversely, this was one of the key benefits of the F2F Health Check. This was perceived as a crucial part of the Health Check, as individuals want to be reassured that their health is given the utmost standard of care. This was also seen through the interviews as some SUs and HCPs worried that not physically seeing individuals in-person may potentially miss underlying conditions that are not part of the DHC screening. A qualitative study with GPs found that one of the key concerns with using digital, artificial intelligence systems with patients was losing the doctor-patient relationship.^28^ Effective communication between SUs and HCPs is crucial for the provision of care and recovery.^29^,^34^ Many staff and SUs mentioned that they preferred an F2F appointment when discussing results and advice. A key factor to the successful implementation of technological interventions in healthcare is that it helps facilitate discussions with patients.^12^ Previous research on digital healthcare, particularly for mental health services where the doctor-patient relationships are vital, found this lack of interaction with HCPs in the digital sphere a big challenge.^35^,^37^ This element of care is absent from the current DHC service.

HCPs and SUs both expressed some form of concern surrounding the accuracy of the physical tests if they are completed by someone who is not an HCP. Doing these measures may be considered a high-stakes activity that will impact health results and thus some users indicated that they would prefer a professional to do the tests for them. Also, involving third-party providers for physical tests presented a challenge in collating all updated metrics back into the system for the user. SUs are concerned that this needs to be fed back to the GP, so they can assess the level of risk. This adds to the points made in the previous paragraph about F2F communication being an important factor for patient care, and some SUs need the reassurance of HCPs to be confident in their results and next steps.

Finally, only one HCP who was interviewed had experience with the DHC, indicating a clear lack of awareness and understanding of the service. All practices involved in the study area were sent interview invitations and would have been expected to be aware of the DHC. This awareness of the programme was not seen with the interviewed HCPs; perhaps, this indicates poor communication within practices potentially between management and staff. Regardless, this had an impact on the acceptance and trust of the idea of the DHC service among interviewed staff. Furthermore, from the single HCP who was aware of the service, there appeared to be a disconnect between the F2F Health Check patient record system and the DHC system, which led to additional work for the HCP. It is unclear whether this was a failure of the system or a lack of understanding on the part of the HCP. The potential disconnect between the F2F Health Check record system and the DHC system was a concern echoed by staff and SUs alike. These findings are supported by a systematic review conducted on the facilitators and barriers to implementing technological interventions in healthcare.^12^ The review found that if staff perceive the intervention to increase workload, cause disruption and need additional staff members, this acted as a barrier to implementation. Facilitators were factors such as adequate training, pilot testing, links to relevant clinical and patient information, endorsement from senior peers, and if the system supported a known organisational challenge.^12^ These facilitators should be taken into account in the future implementation of DHC programmes.

The strengths of the study are the focus on SUs’ real experience of the DHC and the F2F Health Check and the provision of new information about innovation in healthcare practice. This study is limited by a smaller number of interviews with HCPs than intended. We faced difficulties recruiting HCPs who had experience with the DHC being used in their practice and who had experience with patients who had completed the DHC. As a result, this may present a limited view of the DHC as other HCPs interviewed expressed their assumptions as opposed to real-life experiences of the service. The majority of SUs interviewed were of white ethnicity, which may affect the generalisability of the findings. Finally, the period of time between when the Health Check was undertaken, and the interviews may have presented with difficulty in recalling the experience.

Overall, there is a need for a digital solution to address the demand and pressure within GP clinics. In its current form, the DHC has benefits and barriers to its use according to both HCPs and SUs. The DHC appears to be acceptable for lifestyle questions but not for physical tests due to concerns surrounding accuracy, confidence and removing the apparent convenience of the DHC. To improve the implementation of the DHC in the future, the following recommendations have been suggested based on the study findings: communicate problematic results and advice in person, provide an opportunity for discussion, and raise awareness among HCPs of the DHC as a complementary service to the F2F Health Checks and its potential to address the challenges experienced by GP clinics. These recommendations may increase the acceptability of the DHC overall and facilitate its implementation in the healthcare system.

## supplementary material

10.1136/bmjopen-2024-090492 online supplemental file 1

10.1136/bmjopen-2024-090492 online supplemental file 2

## Data Availability

Data are available upon reasonable request.
