# Supplementary material for: Exploring service users’ and healthcare professionals’ experience of digital and face-to-face Health Checks in England: a qualitative study
Source: BMJ Open. 2025 Mar 13;15(3):e090492. doi: 10.1136/bmjopen-2024-090492 (PMC11907040; doi:10.1136/bmjopen-2024-090492)
Supplement: online supplemental file 1 [file bmjopen-15-3-s001.docx]

**Supplementary Materials 1: SRQR Checklist**

**Standards for Reporting Qualitative Research (SRQR): a synthesis of recommendations**

All topics and numbers of this checklist are directly cited from Table 1 in: O'Brien BC, Harris IB, Beckman TJ, Reed DA, Cook DA. Standards for reporting qualitative research: a synthesis of recommendations. Acad Med. 2014;89(9):1245-1251.

|  | Topic | Part of manuscript information can be found | Page numbers* |
| --- | --- | --- | --- |
| 1 | Title | Title | 1 |
| 2 | Abstract | Abstract | 2 |
| 3 | Problem formulation | Introduction | 4-6 |
| 4 | Purpose or research question | Introduction | 6 |
| 5 | Qualitative approach and research paradigm | Methods | 9 |
| 6 | Researcher characteristics and reflexivity | Methods | 9 |
| 7 | Context | Methods | 7 |
| 8 | Sampling strategy | Methods | 7 |
| 9 | Ethical issues pertaining to human subjects | NA |  |
| 10 | Data collection methods | Methods | 7 |
| 11 | Data collection instruments and technologies | Methods | 8 |
| 12 | Units of study | Results | 10-16 |
| 13 | Data processing | Methods | 9 |
| 14 | Data analysis | Methods | 9 |
| 15 | Techniques to enhance trustworthiness | NA |  |
| 16 | Synthesis and interpretation | Results | 10-16 |
| 17 | Links to empirical data | Results | 10-16 |
| 18 | Integration with prior work, implications, transferability, and contribution(s) to the field | Discussion | 16-19 |
| 19 | Limitations | Strengths and limitations | 19 |
| 20 | Conflicts of interest | Competing interests | 20 |
| 21 | Funding | Funding | 20 |

*All page numbers refer to pages in the submitted manuscript file

**Supplementary Materials 2: Original Project Protocol**

(Submitted as a separate file)

**Supplementary Materials 3 - Interview Schedule for Service Users**

**Patient experience of the Southwark NHS Health Check service – interview schedule**

Thank you for agreeing to take part in this project. As you’ll have read in the information document, we are interested in your experience and views of the new Health Check service in Southwark. So, in this call, I’ll ask you some questions about this – it does not matter if you did not complete a Health Check and there are no right or wrong answers, we’re just interested in your opinions. I’ll record what is said in this call so that it can be typed up later, but any information that could identify you, such as names of people or places, will be removed. If at any time during the call you would like to stop, just let me know, and you do not have to answer any questions that you do not feel comfortable with.

Before we begin, do you have any questions for me?

[Start recorder]

To start, can you tell me if you completed a Health Check, either online or at the GP practice?

[If yes – go to page 2]

[If no]

Can you remember receiving an invitation for a Health Check?

[If yes] Did you receive an text message or letter invitation? How would you prefer to receive an invitation?

What did you think when you received this invitation?

Did you receive any reminders? (Text or letter?) And what did you think of these?

Did you try to book a Health Check? Why/why not?

[If booked but not completed] What stopped you from completing/attending the Health Check?

[If no] The Health Checks aim to identify people who are at higher risk of developing long term health problems so that they can be offered help to lower their risk, for example, support with becoming more physically active or quitting smoking, or being prescribed medication. Is that something that you would be interested in?

How would you like to receive an invitation to a Health Check (text/letter)?

For those who did complete a Health Check]

What made you want a Health Check?

Did you choose the online Health Check or a face-to-face one at your GP practice?

Why did you choose this option?

Tell me about your experience of your Health Check, from booking/logging on to getting the results.

[F2F prompts] Booking appointment – convenient time? Waiting?

Consultation – assessments completed? Rapport with GP/nurse?

Results – in appointment or later? Follow up advice – able to ask questions?

[DHC prompts] Navigating site – any difficulties? Were the questions easy to answer/understandable?

Did they book a physical assessment? Tell me about this (booking, place, consultation)

Results – how received? Follow-up advice – able to ask questions?

Did your Health Check prompt you to make any changes or look for more information on your health?

[If yes] What did you look into/changes have you tried? (e.g., PA, diet, smoking, medication)

How are you getting on with [the behaviour change]?

Would you recommend that a family member or friend of a similar age to you had a health check if invited?

If yes or no probe why

Finally, is there anything that would make the Health Check service better for you, either in terms of how it was conducted or what happened afterwards?

That’s all my questions, is there anything else you would like to add?

Thank them for their time and stop recorder.

**Supplementary Materials 4 - Interview Schedule for HCPs**

**HCP experience of the Southwark NHS Health Check service – interview schedule**

Thank you for agreeing to take part in this project. As you’ll have read in the information document, we are interested in your experience and views of the new Health Check service in Southwark. So, in this call, I’ll ask you some questions about these – there are no right or wrong answers, we’re just interested in your opinions. I’ll record what is said in this call so that it can be typed up later, but any information that could identify you, such as names of people or places, will be removed. If at any time during the call you would like to stop, just let me know, and you do not have to answer any questions that you do not feel comfortable with.

Before we begin, do you have any questions for me?

[Start recorder]

To start, can you tell me how long you have been a practice nurse/healthcare assistant/practice manager?

How much experience would you say you have with conducting Health Checks? (N.B. they started in 2009)

Can you tell me how you conduct the standard face-to-face Health Check appointments?

What preparation, if any, would you do before the appointments?

[*for nurses/HCAs*] How do you deliver advice/information to patients following the assessments? (signposting/leaflets, how long does this take?)

Following the appointment, what extra tasks are involved for you or your colleagues? (e.g., to record results, organise follow-ups)

And now please can you tell me how you have found the addition of the digital Health Checks to the service?

What work is involved for you and your colleagues when patients choose to complete a Health Check online?

Has there been an impact on numbers of patients seen in person for Health Checks?

[*for nurses/HCAs*] How have you found seeing people who have been identified as high risk after they have completed an online Health Check, in comparison to seeing them for the full Health Check?

Do you have all the information you need from the digital results or is extra questioning needed?

What, if anything, do you consider to be the benefits of providing digital as well as face-to-face Health Checks?

And what, if anything, are the risks?

Are there any improvements or changes that you would like to see made to the Health Check service?

Overall, do you think the addition of digital Health Checks to the service is a good idea/should be rolled out further?

Are there any other comments that you would like to make about health checks, in person or face to face?

That’s all my questions, is there anything else you would like to add?

Thank them for their time and stop recorder.

**Supplementary Materials 5 – Revised Analytical Framework (Service Users)**

| **Theme** | **Sub-themes** |
| --- | --- |
| Service user demographics | Age  Gender  Ethnicity  Health Check type (digital/F2F/none)  Education level  Employment status |
| Invitation and booking | Understanding of the Health Check (awareness, what it is for etc)  Invite method (text, letter etc) (and initial response to this, preference)  Reminders?  Choice of F2F/digital (option?/reasons for choosing)  Booking process for F2F (ease, challenges etc)  Starting digital Health Check (timing, ease of process etc)  Other |
| Motivation | Perceived health  Understand more about health  Previous medical background  Personal responsibility  Prevention  NHS cares (the organisation is looking after me etc)  Other |
| F2F Health Check experience | Questions during Health Check  Person completing Health Check (profession, manner)  Physical tests? (yes/no/what)  Timing and communication of results  Asking questions  Advice given (services, signposting etc)  Benefits of F2F Health Check  Other |
| DHC experience | Website (understanding/navigation/ease of use etc)  Benefits (convenience, personality)  Digital age  Physical tests (any, how/where these happened etc)  Barriers/problems (digital technology; doing tests, asking questions, conflicting advice etc)  Timing and communication of results  Advice given (referrals, services etc)  Other |
| Behaviour change | Changes made (what, why, include services attended)  Maintenance  If none, why  Impact of changes  Other |
| General | Recommend Health Check (and why)  Improvements to Health Check (digital and F2F)  Preference for digital/standard (why, belief that everything going online etc)  Other |

**Supplementary Materials 6 Revised Analytical Framework (HCPs)**

| **Theme** | **Sub-themes** |
| --- | --- |
| Experience | Nurse/role experience (years etc)  Experience with Health Check previous (training, confidence)  Other |
| Conducting F2F Health Checks | Prepare before (and experience/opinions of this)  Running the health check (general)  Giving advice (signposting, delivering it, experience of it, time etc)  Admin after (and experience/opinions of this)  Other |
| Digital Health Checks | Understanding of them (awareness etc)  Additional work (pre, post, follow up?)  Increase in DHC attendees? (and opinions on this)  DHC vs standard for high risk people (experience of this, does it work, benefits, negatives etc)  Benefits of DHC (choice, convenience, workload etc)  Risks of DHC (honesty, tech issues, miscommunication, results)  Health Check improvements?  DHC rollout opinion  Other |

**Supplementary Materials 7: Example of the DHC results page– Page 1**

**
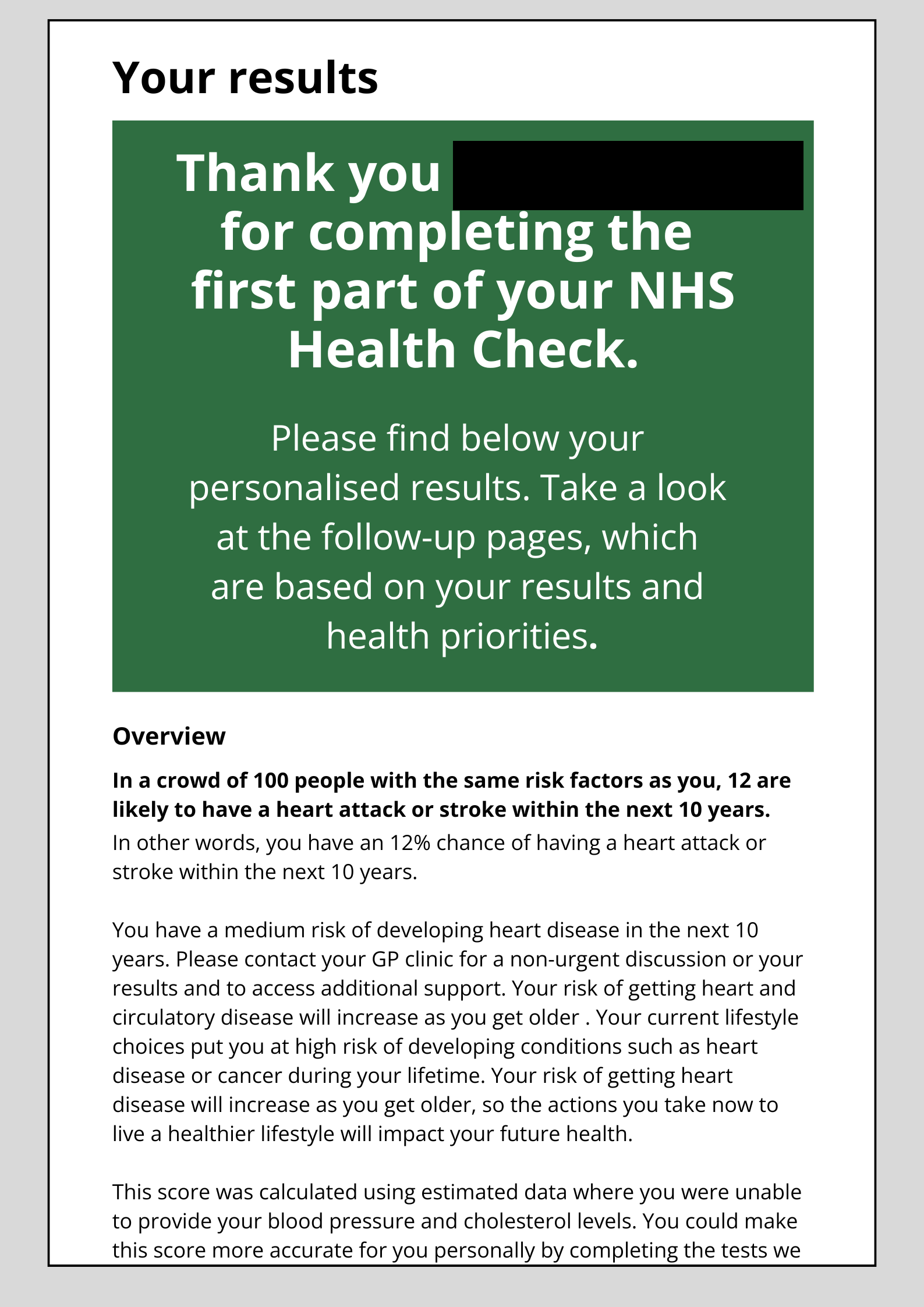
**

**
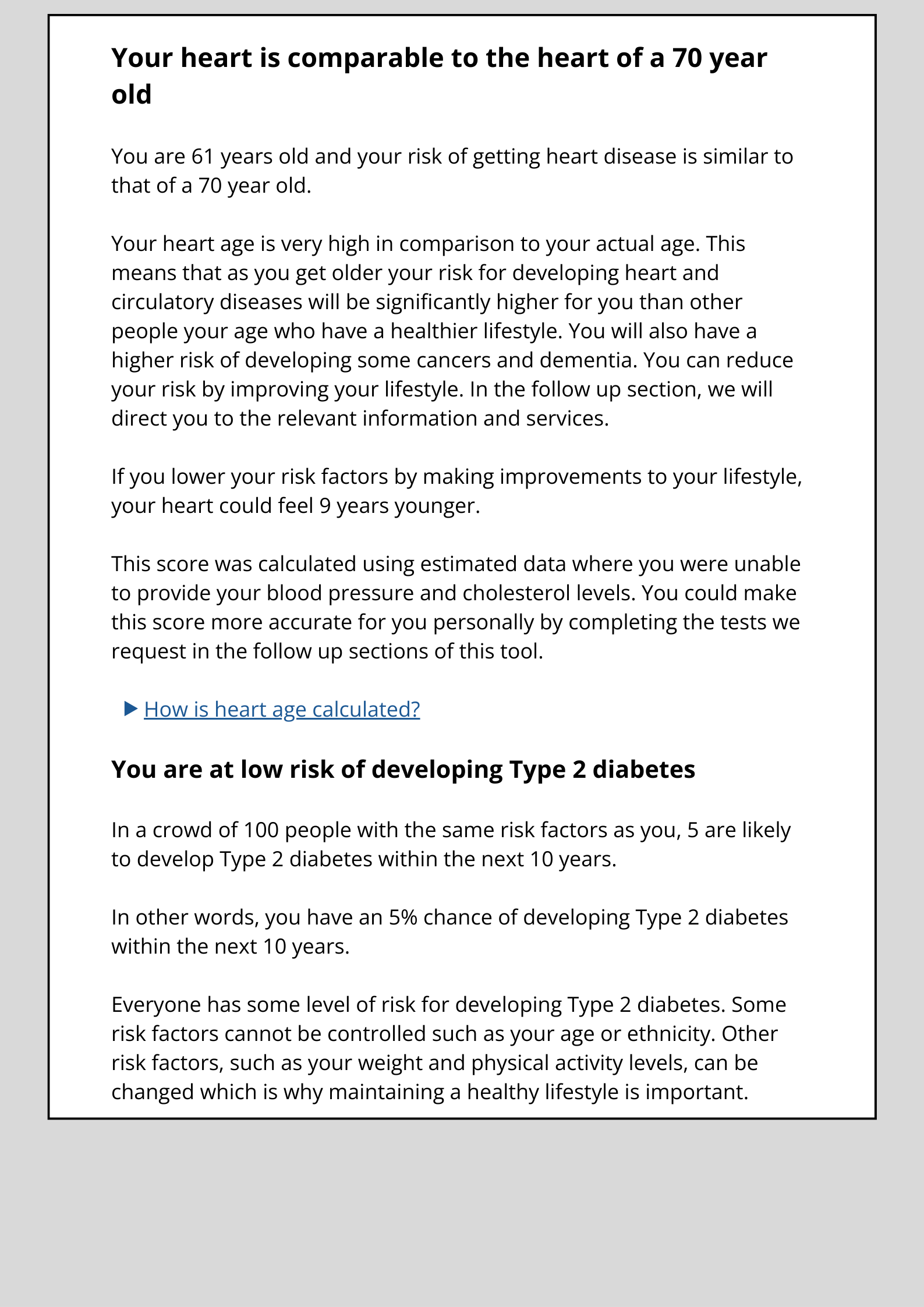
 Supplementary Materials: Example of the DHC results page – Page 2**

**Supplementary Materials: Example of the DHC results page– Page 3**

**
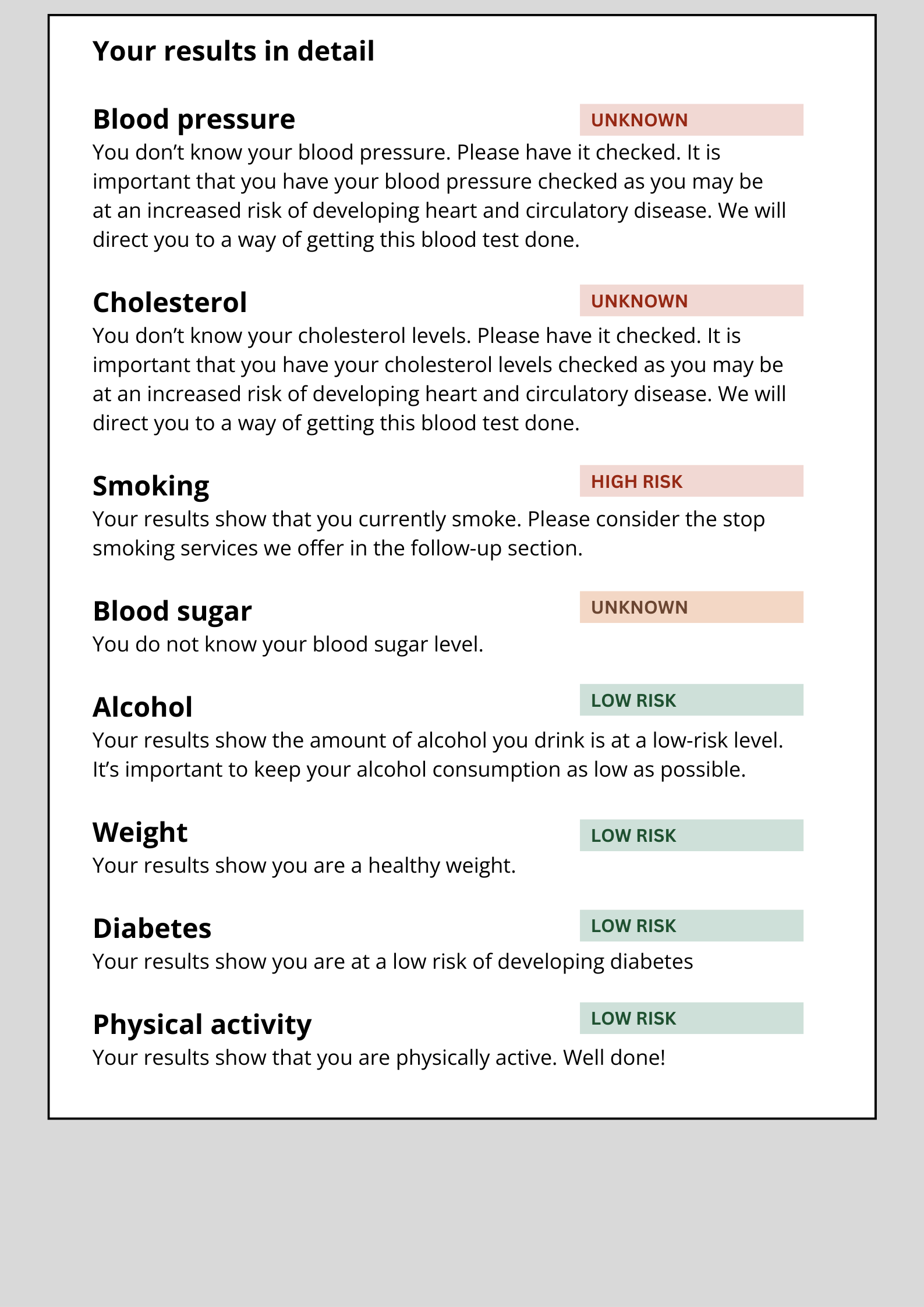
**
