# Supplementary material for: Exploring service users’ and healthcare professionals’ experience of digital and face-to-face Health Checks in England: a qualitative study
Source: BMJ Open. 2025 Mar 13;15(3):e090492. doi: 10.1136/bmjopen-2024-090492 (PMC11907040; doi:10.1136/bmjopen-2024-090492)
Supplement: online supplemental file 2 [file bmjopen-15-3-s002.docx]

PHIRST Insight

Protocol for the evaluation of the Southwark NHS Health Check service

Version 3

NHS REC approval received 21/12/2022: reference 22/EM/0280

| Funder | NIHR PHIRST |
| --- | --- |
| Chief investigator | Prof Russ Jago, PHIRST INSIGHT, University of Bristol |
| PHIRST Team | Dr Lis Grey (Senior Research Associate), Prof Rona Campbell, Prof Frank De Vocht (Quantitative analysis) and Dr Hugh McLeod (Health Economics) (all based at the University of Bristol/NIHR ARC West) |
| Southwark Team | Paul Stokes (Head of Programme – Health Improvement), Jin Lim (Deputy Director of Public Health) and Cris Amankwah (Head of Digital Public Health) |
| Public and Practice stakeholders | Modupe Alimi, Patrick Erhiakporeh (local residents, Southwark Healthwatch ambassadors)  Linda Drake (practice nurse in Southwark), Payam Torabi (GP, formerly practising in Southwark, now based in Tower Hamlets) – both were involved in the development of Southwark’s digital Health Check |
| Timeline | September 2022 – May 2024 |

# Background

The NHS Health Check programme aims to detect early signs of cardiovascular disease (CVD), type 2 diabetes (T2DM), kidney disease, stroke and dementia in 40 to 74 year olds in England (1). Adults meeting the eligibility criteria for a Health Check are invited to attend a face-to-face appointment in their GP practice, where they are assessed for the top seven risk factors of non-communicable diseases (NCDs): physical inactivity, excess weight, tobacco smoking, excess alcohol consumption, high blood pressure, high cholesterol, impaired glucose processing. Behavioural support and, if appropriate, pharmacological treatment or further tests may then be offered to help an individual reduce their risk of disease. As well as promoting early identification and management of behavioural and physical risk factors, the Health Check programme is intended to reduce inequalities in the prevalence and burden of behavioural risk factors and NCDs. Local authorities are responsible for the commissioning and delivery of the Health Check programme and have some flexibility in this, however, to help ensure the quality and safety of the programme, the measurements conducted and actions to be taken in response to certain risk factors are standardised (1). The Office for Health Improvement and Disparities (OHID, formerly Public Health England) aspires to achieve a national uptake rate of 75% of the eligible population having a Health Check once every five years (1); to monitor progress towards this goal, all local authorities submit data to central government on the Health Checks offered and received each quarter. An evaluation of the Health Check programme delivered between 2012 and 2017 reported an average uptake rate of 52.6% across England (2), however, since 2018, the uptake trend has been decreasing (3).

The London borough of Southwark has significant health inequalities with residents from the more deprived central areas living on average seven years less than those from the least deprived areas. At least 70% of adults in Southwark have two or more behavioural risk factors for preventable NCDs, with prevalence being greater among the more deprived communities. Looking at their Health Check data for the years 2017 – 2020, Southwark Council identified low uptake among certain groups in their population, namely those in the most deprived quintile (IMD Q1; 53% of those invited from this group attended), those aged 40-44 years (46% attendance) and men (46% attendance). These rates were despite targeting invitations at men and those living in the most deprived areas. To help reach these groups and increase impact of the Health Check programme, Southwark Council has developed a Digital Health Check (DHC), which eligible patients can complete online at a place and time convenient to them. The DHC operates like an online survey, incorporating the CVD QRisk3 and QDiabetes screening questionnaires, where users answer a series of questions about their health and behaviours, as they would in a face-to-face Health Check. Following completion of the DHC, users are invited to complete physical health assessments (e.g., blood pressure measurements) at either a community pharmacy or sports centre. The results from the DHC and physical assessments are sent to the individual’s GP practice and, if early signs of disease are detected, they will be invited to attend a face-to-face appointment. If risky health behaviours or weight are detected in the DHC without early signs of disease, individuals are signposted to sources of support for adopting healthier behaviours or reducing their weight. Thus, for those reticent or less able to attend the standard face-to-face Health Check, the DHC process may present a more acceptable alternative that still enables the delivery of preventive advice and the identification of early-stage disease.

The DHC underwent final stage beta testing in primary care this February (2022) and Southwark Council plan to roll out the DHC alongside the standard face-to-face Health Checks in a pilot trial in the north and central regions of Southwark (4 GP neighbourhood areas - Bermondsey, Borough, Rotherhithe and Walworth) running from January to March (inclusive) 2023. Invitations will offer patients the choice of completing either a digital or face-to-face Health Check, thus those who wish to complete their Health Check at their GP practice will still be able to do so. The PHIRST team at the University of Bristol have been asked to help evaluate Southwark’s Health Check service. Through the evaluation we will seek to understand:

1. The extent to which the DHC is effective at engaging those groups that have not been reached by the standard Health Check
2. Whether the service overall is effective at encouraging people to take positive health actions and how the service could be improved
3. If effectiveness differs among those completing the DHC versus a face-to-face Health Check
4. Practice nurses’ (PNs), healthcare assistants’ (HCAs) and GP practice managers’ (PMs) perceptions and experiences of both the standard and digital Health Checks and the impact on GP practices of the addition of DHCs, in terms of clinician and administrative burden.
5. The cost of the DHC as business as usual and whether it represents good value.

# Logic model

#
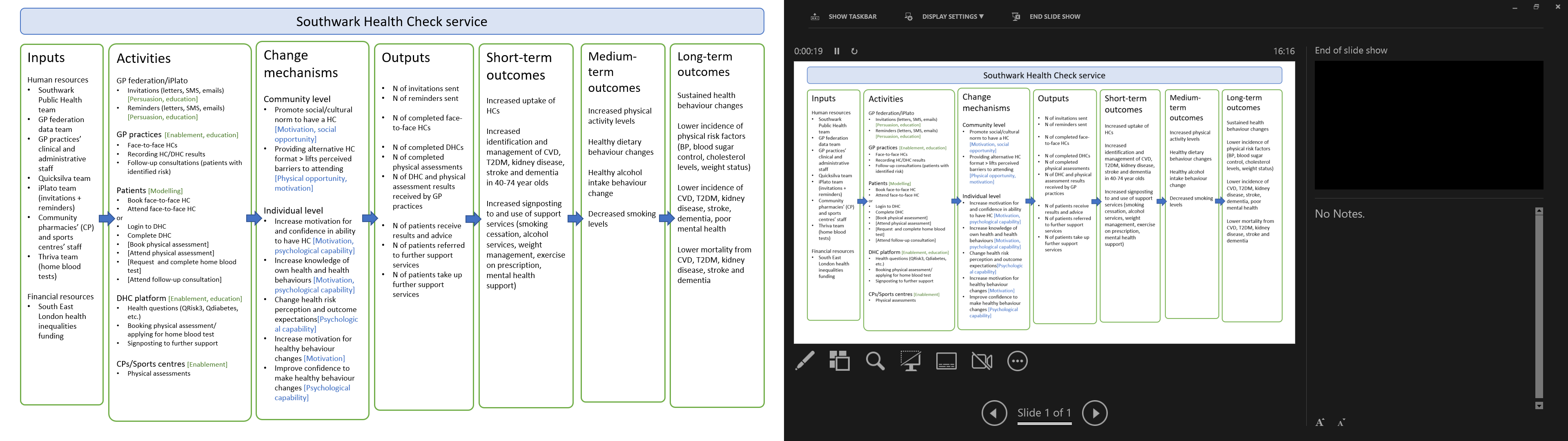


# Literature review

The NHS Long Term Plan (4), published in 2019, set out a vision for ‘digital transformation’ with the broad aims of improving patients’ experience of services and increasing efficiency. NHSX was set up to drive this digital transformation, having responsibility for setting policy, overseeing implementation and allocating investment. Priority areas for NHSX (5) include: enabling safe and secure flow of digital information between care settings; creating straightforward digital access to NHS services to help patients manage their health; using digital tools to personalise services and better target eligible populations to improve service uptake; and using digital tools to ‘capture data as a by-product of care’, reducing administrative burden while improving the ability to plan services. Primary care services are to be a forerunner in the digital transformation, with a ‘digital-first’ delivery model set to be in place by 2023/24 in which all patients will have the right for their first point of contact with primary care professionals to be through digital channels (4). The ‘Plan for digital health and social care’ (6), published in 2022 by the DHSC, further specified aims that the NHS in England would have digital health checks and risk-based screening by 2028.

A rapid evidence synthesis conducted in 2018 to inform NHS England policy on digital-first primary care (7), reported several potential advantages to offering alternatives to face-to-face care delivery, including providing more control and convenience to patients, particularly those with decreased mobility. Remote consultations were also thought to be preferable to patients who are apprehensive about face-to-face medical encounters; digital communication giving a greater feeling of privacy, which may overcome sociocultural barriers such as embarrassment and stigma around health seeking behaviours. However, where digital and other forms of remote care were offered, they tended to be used by younger people, women, those with English as their first language and those with higher incomes and education levels. These findings raise concerns that a shift to more digital and remote delivery may increase health inequalities by further limiting access to older adults and socioeconomically disadvantaged groups. Clinicians also expressed concern that important cues and symptoms may be missed in remote delivery, which could help explain findings that GPs engage in more ‘safety-netting’ practice (such as inappropriate antibiotic prescribing) when they cannot see a patient face-to-face. There was some evidence to suggest that digital triage tools could divert demand away from primary care services, enable greater flexibility in working schedules for practitioners and provide cost savings compared to standard care, but results varied between interventions and outcome measures. The authors also highlighted a need for studies on the number and duration of follow-up consultations after digital consultations to fully assess impact on workload. Poor infrastructure and lack of staff training in digital services delivery within the NHS were reported as further barriers. Overall, there was little high-quality evidence available to include in the review and a particular lack of empirical data to compare the benefits and risks of digital services with standard, face-to-face primary care.

The evidence synthesis by Rodgers and colleagues was conducted prior to the COVID-19 pandemic. To provide care amid the social distancing restrictions implemented to prevent the spread of COVID-19, public, primary and secondary health services rapidly reconfigured to deliver care remotely (8, 9). This shift was largely well-received by both clinicians and patients (10), and will likely have improved acceptance of and skills with using technology for healthcare. However, while digital technology undoubtedly helped during the peak of the pandemic, the digital delivery models implemented during this period will need to evolve now restrictions have lifted. Recent research has highlighted primary care clinicians’ ongoing concerns over the increased clinical risk involved in remote care, as well as variable levels of skills and confidence in using technology among primary care staff (10). This suggests greater guidance and training over when and how to use technology is needed. Patients’ expectations for how they receive health services are also likely to have changed now restrictions have lifted and different delivery modes are available (11). Furthermore, while access to digital healthcare may have increased for the population overall, inequalities in access may have increased as those in the lowest income groups, or who are living with physical or mental disabilities, or whose first language is not English are still likely to face digital exclusion (12). Questions have also been raised over the sustainability, costs and implications for provider workloads of digital delivery models; there is some evidence to suggest that increasing access through offering digital services can lead to supply-induced demand but further research is urgently needed to better understand these factors (13).

# Evaluation aims and objectives

### Aims

This evaluation of Southwark’s combined Health Check service aims to understand who completes the digital and face-to-face offers, why they chose either the digital or face-to-face option (or to not take up either), what they think of the service and whether it has had any impact on their health behaviours. We will also explore the costs involved in both offers and the impact on primary care providers (practice nurses (PNs), healthcare assistants (HCAs) and practice managers (PMs)) of adding digital Health Checks to the standard service.

### Objectives

1. Assess the extent to which the DHC is effective at engaging those groups that have not been reached by the standard Health Check
2. Explore to what extent the service overall is effective at encouraging people to take positive health actions and how the Health Check process could be improved
3. Explore whether effectiveness, in terms of encouraging positive health actions, differs among those completing the DHC versus a face-to-face Health Check
4. Explore PNs’/HCAs’ and PMs’ perceptions and experiences of both the standard and digital Health Checks and the impact on GP practices of the addition of DHCs, in terms of clinician and administrative burden
5. Investigate the cost of the DHC as business as usual and assess whether it represents good value.

# Methods

## Design

This will be a mixed methods project that will include the following methods to address each Research Objective (RO).

1) Quantitative analysis of pseudonymised patient data and DHC analytics data (RO1). Pseudonymised patient data will be compared for all patients sent invitations to standard and digital Health Checks throughout quarter four (Jan – Mar 2023).

2) Patient survey conducted 6-months after the Health Check invitations (both standard and digital Health Check invitations) (RO1, RO2, RO3)

3) Semi-structured interviews with patients following completion (or non-completion) of the Health Checks (RO2, RO3)

4) Semi-structured interviews with PNs, HCAs and PMs (RO4)

5) Economic analysis of time and resource costs (RO5)

Figure 1 details the study methods, along with the research objectives they are intended to address, and expected timings.


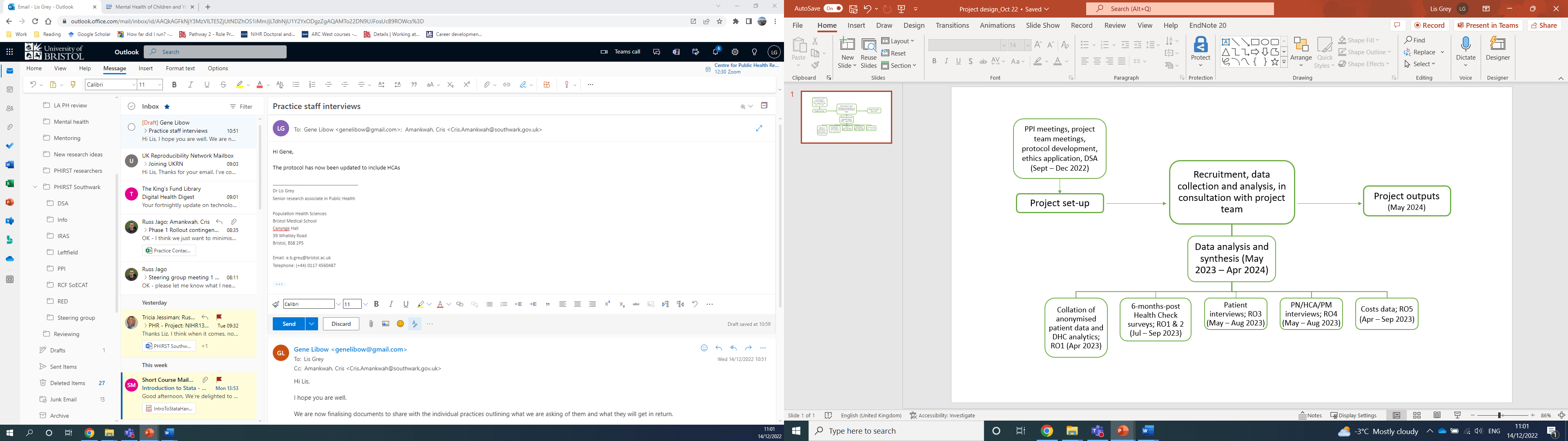


Figure 1. Overview of evaluation methods and timeline

This research protocol has been co-produced with our key partners. The protocol has been developed with input from PPI group members (recruited through community groups in Southwark), local GPs and PNs (key stakeholders) and the project team (consisting of the two PPI representatives, local GP and PN representatives, the Southwark public health team and the University of Bristol PHIRST team). We will continue to work with our PPI group throughout the project to refine recruitment strategies and research materials, and to develop a Dissemination, Impact, Involvement, Communication and Engagement (DIICE) plan to guide the outputs from this work.

## Participants and recruitment

This project will involve patients in north and central Southwark (4 GP neighbourhood areas – Bermondsey, Borough, Rotherhithe and Walworth), an area with high levels of deprivation and a population of diverse ethnicities. The eligibility criteria for a Health Check, as set out in the NHS Health Check Best Practice Guidance (1), are:

- aged 40 – 74 years
- registered with a Southwark GP
- not had a Health Check in the previous 5 years
- not registered as having any of the following: coronary heart disease; chronic kidney disease (CKD), which has been classified as stage 3, 4 or 5; diabetes; hypertension; atrial fibrillation; transient ischaemic attack; familial hypercholesterolemia; heart failure; peripheral arterial disease; stroke
- not receiving palliative care
- not currently being prescribed statins for the purpose of lowering cholesterol
- not been found (either in a previous Health Check or any other health service in England) to have a 20% or higher risk of developing CVD over the next ten years.

Among those eligible for a Health Check, patients are prioritised to receive a Health Check invitation according to the following criteria (in order): those who have not received a Health Check invitation in the past 6 months are prioritised; those belonging to a Black, Asian or ethnic minority group are prioritised; and those at higher risk for developing a cardiovascular disorder or type 2 diabetes are prioritised. Patients’ risk is estimated from existing information in their health records, such as whether they have a BMI over 30 or are on a prediabetes register. The same algorithm for identifying high risk patients among the Health Check-eligible population will be used to identify patients to receive both standard and digital Health Check invitations.

In quarter four, 6000 people will be invited for a digital Health Check in the central area (the digital Health Check will only be available to people in the central area of Southwark during this evaluation). Invitations to digital Health Checks will be sent by SMS or letter, depending on the contact information the GP federation holds for the patient, and contain a link to the digital Health Check website. The invitations also state that patients can book a standard Health Check at their GP practice if they would prefer. In quarter four, approximately 3000 invitations for standard Health Checks will be sent in the study area^[[1]](#footnote-2)^. Invitations for standard Health Checks are also sent by SMS or letter and only offer patients a Health Check at their GP practice. All invitations (for both standard and digital Health Checks) are sent on behalf of the GP federation by iPlato, a healthcare technology company. The same algorithm will be used to identify patients to be sent a digital Health Check invitation as that used for standard Health Check invitations.

### Survey recruitment

Within the central area, all patients receiving an invitation in quarter four (Jan – Mar 2023) to complete either a standard or digital Health Check will be sent invitations to complete an online survey six months after their original Health Check invitation (i.e., Jul – Sep 2023). Invitations, and up to 2 reminders, will be sent by iPlato via SMS or letter (according to information available on the patient’s records) and direct the reader to an online survey. Survey completion will be optional and submission of a survey will be taken as consent for the individual’s data to be used for research purposes. At the start of the survey, patients will be notified of this and presented with information on the purpose of the survey and how their data will be used (i.e., for the purpose of research and service improvement). Patients taking part in the survey will be offered entry into a prize draw to win one of ten £50 shopping vouchers.

### Patient interview recruitment

An external market research company, Leftfield, will recruit participants for the patient interviews. An invitation will be sent by iPlato via SMS to all patients who were invited to a Health Check (both digital and standard) between January and March 2023. Interview invitations will provide a link to the participant information sheet and a link to an online form where individuals can provide their consent and contact details and answer some demographics questions in order to be screened and contacted by Leftfield. We aim to recruit approximately 30 patients for interviews. Leftfield will screen volunteers to ensure the sample includes a range of people according to gender, age and ethnic groups, area of residence, and Health Check completion status (i.e., completion of the standard Health Check, the digital Health Check or did not complete a Health Check). Leftfield will then phone participants to check they understand what is involved in participating and are still happy to be interviewed; if so, Leftfield will send them an electronic consent form. When consent has been given, Leftfield will arrange telephone/online interviews between participants and a Bristol-based researcher.

### PN/HCA/PM interview recruitment

All GP practices in the central Southwark area will be sent invitations for one of their PNs/HCAs or PMs to take part in an interview. Invitations will be sent on behalf of the University of Bristol by the Southwark public health team and direct the PN/HCA/PM to an online form where a full participant information sheet will be available to read and download before completing a consent form, a demographic survey and a contact details form. The Bristol team will then contact the PN/HCA/PM to arrange a suitable time for an interview. We aim to recruit about 10 PN/HCA/PMs for interviews, representing a range of practice sizes.

## Procedure

Pseudonymised patient data will be collated from patients’ records (held by the GP Federation) and shared with the University of Bristol team. The digital Health Check website developer (Quicksilva) will provide analytics data on website usage to Southwark, which will be shared with Bristol. The patient surveys will also be run by the Southwark team and pseudonymised results shared with Bristol. Surveys will contain questions on patients’ health behaviours and actions taken following their receipt of a Health Check invitation.

Interviews for both patients and PN/HCA/PMs will take place via telephone or videocall, according to participant preference. Interview schedules for patient and PN/PM interviews were co-developed by the whole project team, with input from the PPI group. Briefly, patient interviews will seek to understand why patients chose either a standard face-to-face Health Check or digital Health Check or neither, what was their experience of the service and in what ways the service could be improved. Interviews with PN/HCA/PMs will cover their experience of providing the combined Health Check service, including any impact on workload for them and their colleagues, any concerns or perceived benefits of the service, and any suggestions for improvements. The interviews will be semi-structured, allowing the researcher to adapt the questioning according to the participant’s earlier responses and prompt for further information if relevant novel issues are raised (14). Participants will have completed an online consent form prior to the interview, but the researcher will check their understanding of the interview procedure and how their data will be used at the start of their meeting. Interviews are anticipated to take about 30 minutes and will be audio recorded using an encrypted digital recorder then fully transcribed verbatim. Participants will be offered a £50 Love2Shop gift voucher for taking part.

Costs data associated with the development, implementation and use of the digital Health Check software will be collated by Southwark and shared with Bristol.

# Analysis

### Quantitative analyses

Descriptive analysis of pseudonymised patient data will be used to develop profiles of completers of face-to-face Health Checks and digital Health Checks as well as non-completers. To assess the extent of differences between the three profiles, comparative analyses (ANOVA, Pearson Chi-square, multiple regression, independent t-test or Mann-Whitney test) will be conducted as appropriate. Analytics data from the digital Health Check website and quantitative survey data will be coded to assess how patients use the service and whether and what changes to their health behaviours they make as a result. Quantitative analyses will be conducted using SPSS/STATA and Excel software.

### Health Economics analysis

The survey of people six months after being invited to a face-to-face or digital Health Check in quarter four of 2022/23 will provide a basis for initial exploration of the incremental impact of the digital Health Check pathway on selected costs and outcomes. For example, the reported action of “Taking prescribed medication (e.g., statins)” to improve cholesterol levels will be costed using national reference cost data for statins (15). Reported general practice attendances will be costed using national reference cost data (16), and costs associated with reports of interventions and tests, such as weight management, will based on literature. We will request data from the Southwark public health team on costs associated with the development, implementation and use of the digital Health Check software. To inform potential future evaluation, we will explore the feasibility of using the survey data and pseudonymised patient data with the workHORSE model to estimate the long term cost-effectiveness of the digital Health Check intervention (17).

### Qualitative analyses

Interview transcripts will be analysed using a thematic Framework approach (14), conducting separate analyses for patient and PN/HCA/PM interviews. After reading all transcripts, a draft coding framework will be developed including themes and sub-themes that are driven by the data but also relevant to our research objectives. The draft framework will be used to code a sub-sample of the transcripts, then reviewed and amended as necessary to ensure the framework captures all the pertinent information for this evaluation. The coding framework will be entered in NVivo software to be applied to all transcripts. Analysis will be an iterative process – the team will regularly review the framework to ensure it is still a good ‘fit’ for the data. When all transcripts have been coded a framework matrix will be developed with columns to represent each sub-theme and rows for each participant. Cells will be populated with quotations, data summaries and researcher’s analytic notes. This ‘charting’ method creates an accessible dataset through which to explore themes and subthemes by respondent type. A summary of the data under each sub-theme will be developed to inform the next stage of the analysis, moving up the analytical hierarchy to explore patterns and associations between themes in the data.

# Data management

The University of Bristol will be the data controller for this study. The project will generate quantitative datasets, in the form of pseudonymised patient data, patient surveys and costs data, and qualitative datasets, in the form of interview transcripts.

All patient record and survey data will be pseudonymised (names, contact details and postcodes will be removed and unique participant ID numbers assigned) by the Southwark team before being shared with the University of Bristol team. Interviews will be recorded on encrypted digital audio recorders. Audio files from interviews will be uploaded to a restricted access folder on the University of Bristol server, as soon as is reasonably possible following an interview. Once uploaded, they will be securely deleted from recorders. Transcription will be undertaken by an external transcription company that has been approved to process data subject to the Data Protection Act, for which the University is the data controller. The company has entered into a formal "Personal Data Processing Agreement" drawn up by the University Secretary's Office. The University of Bristol project team, including those who may become part of the team in the future, will have access to the study data and will be able to comment on data at the analysis stage. Access to data will be restricted to these individuals. To enable anonymity, transcripts will have a unique identifier in the filename, which will be replicated on a transcript cover sheet that will also include interview location and anonymised interviewee details. No paper copies of transcripts will be made.

All data analysis will take place on password protected University laptops. No data will be stored on laptops but instead on the University’s secure Research Data Storage Facility (RDSF) accessed via the University VPN.

In accordance with Research Councils UK guidance, all consent forms will be stored securely in electronic form for a period of 10 years. After 10 years, the forms will be deleted from servers.

Anonymised data will not be destroyed following completion of the study but restricted access on reasonable request will be kept available for future research in ‘data.bris’ the University's publicly accessible Research Data Repository. Consent for this will be explicitly sought on participant consent forms.

# Ethics

We do not believe that completing the survey or taking part in an interview will result in distress or discomfort to participants. Participants will be able to stop or pause an interview at any time, without having to give a reason. If, during an interview, a participant appears uncomfortable or upset, the researcher will either ask whether they would like to stop or decide to stop the interview and direct them to appropriate support services. We will be contracting Leftfield (an experienced market research company – leftfield.co.uk) to screen and consent participants to interviews. Leftfield specialise in recruitment to qualitative research and understand the importance of ensuring participants understand and are happy with what participating in research will involve, as well as ensuring people do not feel coerced into participating. The University of Bristol based researcher has particular experience in conducting health-related research interviews.

To minimise burden on participants, the length of the surveys and interview schedules have been kept to a minimum. Surveys can be completed at a time and place convenient to participants. Interviews will be scheduled for convenient times for the participants and will be conducted remotely to avoid travel time/expense.

Members of our PPIE group (Southwark residents, in the Health Check age range) felt the study protocol (including surveys and interviews) and study materials (e.g., information sheets) were appropriate and acceptable.

This project has been reviewed (proportionate review) by East Midlands (Nottingham 1) NHS Research Ethics Committee – it received approval on 21/12/2022 (ref: 22/EM/0280).

# Outputs

We will develop and refine a Dissemination, Impact, Involvement, Communication and Engagement (DIICE) plan with the project steering group throughout the project. This is likely to include:

- Report for Southwark Council detailing study findings and recommendations
- Public-facing report and PowerPoint slides on the study findings for Southwark community groups, partner organisations, PHIRST websites and article for The Conversation
- Peer-reviewed journal article on study findings

# Timeline and milestones

|  | Sep 22 | Oct 22 | Nov 22 | Dec 22 | Jan 23 | Feb 23 | Mar 23 | Apr 23 | May 23 | Jun 23 | Jul 23 | Aug 23 | Sep 23 | Oct 23 | Nov 23 | Dec 23 | Jan 24 | Feb 24 | Mar 24 | Apr 24 | May 24 |
| --- | --- | --- | --- | --- | --- | --- | --- | --- | --- | --- | --- | --- | --- | --- | --- | --- | --- | --- | --- | --- | --- |
| **Ethics preparation and submit to REC** | x | x | x |  |  |  |  |  |  |  |  |  |  |  |  |  |  |  |  |  |  |
| **PPI** | x |  |  |  |  |  |  |  |  |  |  |  |  |  |  |  |  |  | x |  |  |
| **Steering group meeting** |  |  |  | x |  |  | x |  |  | x |  |  | x |  |  | x |  |  | x |  |  |
| **Rollout of digital Health Checks** |  |  |  |  | x | x | x |  |  |  |  |  |  |  |  |  |  |  |  |  |  |
| **Health record and costs data shared** |  |  |  |  |  |  |  | x | x |  |  |  |  |  |  |  |  |  |  |  |  |
| **Patient and PN/HCA/PM interviews** |  |  |  |  |  |  |  |  | x | x | x |  |  |  |  |  |  |  |  |  |  |
| **6-month survey** |  |  |  |  |  |  |  |  |  | x | x | x |  |  |  |  |  |  |  |  |  |
| **Analysis** |  |  |  |  |  |  |  |  | x | x | x | x | x | x | x | x |  |  |  |  |  |
| **Data synthesis and reporting** |  |  |  |  |  |  |  |  |  |  |  |  |  |  |  |  | x | x | x | x |  |
| **Dissemination events** |  |  |  |  |  |  |  |  |  |  |  |  |  |  |  |  |  |  |  | x | x |

# References

1. Public Health England. NHS Health Check Best Practice Guidance: For commissioners and providers. PHE publications gateway number: GW-1198. London, UK: Crown; 2019.

2. Patel R, Barnard S, Thompson K, Lagord C, Clegg E, Worrall R, et al. Evaluation of the uptake and delivery of the NHS Health Check programme in England, using primary care data from 9.5 million people: a cross-sectional study. BMJ Open. 2020;10(11):e042963.

3. NHS Fingertips. NHS Health Check data profile <https://fingertips.phe.org.uk/profile/nhs-health-check-detailed/data#page/1>: NHS Fingertips; 2022 [

4. NHS England. NHS Long Term Plan. <https://www.longtermplan.nhs.uk/>: NHS England; 2019.

5. NHSX. NHSX Delivery Plan. Available from: <https://transform.england.nhs.uk/digitise-connect-transform/nhsx-delivery-plan/2021>.

6. Department of Health & Social Care. Policy paper: A plan for digital health and social care. <https://www.gov.uk/government/publications/a-plan-for-digital-health-and-social-care/a-plan-for-digital-health-and-social-care#section-2-our-vision-for-a-digital-future2022>.

7. Rodgers M, Raine G, Thomas S, Harden M, Eastwood A. Informing NHS policy in ‘digital-first primary care’: a rapid evidence synthesis. Health Services and Delivery Research. 2019.

8. Budd J, Miller BS, Manning EM, Lampos V, Zhuang M, Edelstein M, et al. Digital technologies in the public-health response to COVID-19. Nature Medicine. 2020;26(8):1183-92.

9. Gilbert AW, Billany JCT, Adam R, Martin L, Tobin R, Bagdai S, et al. Rapid implementation of virtual clinics due to COVID-19: report and early evaluation of a quality improvement initiative. BMJ Open Quality. 2020;9(2):e000985.

10. Murphy M, Scott LJ, Salisbury C, Turner A, Scott A, Denholm R, et al. Implementation of remote consulting in UK primary care following the COVID-19 pandemic: a mixed-methods longitudinal study. British Journal of General Practice. 2021;71(704):e166.

11. Gilbert AW, Jones J, Stokes M, May CR. Patient, clinician and manager experience of the accelerated implementation of virtual consultations following COVID-19: A qualitative study of preferences in a tertiary orthopaedic rehabilitation setting. Health Expectations. 2022;25(2):775-90.

12. Patient coalition for AI DaDTiH. Putting patients first: Championing good practice in combatting digital health inequalities. Available from: <https://www.patients-association.org.uk/Handlers/Download.ashx?IDMF=a0e5b459-388a-4825-86d6-77910882633c2022>.

13. Salisbury C, Quigley A, Hex N, Aznar C. Private Video Consultation Services and the Future of Primary Care. J Med Internet Res. 2020;22(10):e19415.

14. Ritchie J, Lewis J, McNaughton Nicholls C, Ormston R, Lewis J, McNaughton Nicholls C, et al. Qualitative research practice : a guide for social science students and researchers. Second edition. ed. Los Angeles ;: SAGE; 2014.

15. National Institute for Health and Care Excellence (NICE). British National Formulary (BNF). Available from: <https://bnf.nice.org.uk/2022>.

16. Jones K, Burns A. Unit Costs of Health and Social Care 2021. Personal Social Services Research Unit, University of Kent; 2021.

17. O’Flaherty M, Lloyd-Williams F, Capewell S, Boland A, Maden M, Collins B, et al. Modelling tool to support decision-making in the NHS Health Check programme: workshops, systematic review and co-production with users. 2021;25:35.

1. Invitations for standard Health Checks are limited by the capacity of GP practices, whereas digital Health Checks take up less time and resource for GP practices and so more invitations can be sent. [↑](#footnote-ref-2)
