# Supplementary material for: Exploring service users’ and healthcare professionals’ experience of digital and face-to-face Health Checks in England: a qualitative study
Source: BMJ Open. 2025 Mar 13;15(3):e090492. doi: 10.1136/bmjopen-2024-090492 (PMC11907040; doi:10.1136/bmjopen-2024-090492)
Supplement: Supplementary file 3 [file bmjopen-2024-090492.reviewer_comments.pdf]

PEER REVIEW HISTORY

BMJ Open publishes all reviews undertaken for accepted manuscripts. Reviewers are asked to complete a checklist review form and are provided with free text boxes to elaborate on their assessment. These free text comments are reproduced below.

ARTICLE DETAILS

Title (Provisional)

Exploring service users' and Healthcare Professionals' experience of digital and face-to-face Health Checks in England: A qualitative study

Authors

Forte, Chloe; Grey, Elisabeth B; Jessiman, Patricia; McLeod, Hugh; Salway, Ruth E; Sillero-Rejon, Carlos; Harkes, Rebecca; Stokes, Paul; De Vocht, Frank; Campbell, Rona; Jago, Russ

---

VERSION 1 - REVIEW

**Reviewer**	**1**
**Name**	**Kwan, Bethany M**
**Affiliation**	**Department of Family Medicine, University of Colorado School of Medicine, Aurora, CO, USA.**
**Date**	**03-Sep-2024**
**COI**	**None**

Overall summary and critique: This is a very well written and interesting paper on qualitative analysis of the experience of UK adults (both service users and health care practitioners) with the face to face Health Check and digital Health Check (an entirely digital, asynchronous version of risk assessment) implemented by the National Health Service. The analysis appears rigorously done and the conclusions are appropriate. I have a few minor critiques and suggestions:

1. In the abstract's Objectives section, there is reference to the aim of the study including assessment of "barriers and facilitators to pathways". Then on page 6, it says the aims were to "identify barriers and facilitators to systems". In addition to this inconsistency, it's not clear which systems and pathways are being referred to. Please edit for consistency and clarity.

2. In the abstract, please state how/by whom the participants were recruited.

3. In the introduction, there is a likely typo - extra "ed" after "transferred" on page 4.

4. In the study design, please explain what made this a purposive sample.

5. On page 7 under participant recruitment, there is mention that invitations to complete the Health Check were sent to 9000 people. This is a bigger ask, but I would like to suggest including an analysis of who received the invitations to complete the Health Checks and who actually attended an appointment, including demographics. Especially given the limitations of the qualitative data, the reach of the service (especially who did not participate and whether the sample interviewed was representative of those who used the services) is important to understand.

6. Please add a section on Materials in the methods section and explain the development and content of the interview guides used for both the patient and HCP interviews. Please also specify in the study design section the intended number of interviews for both types of participants.

7. Please clarify, what are "draft thematic frameworks"? Is this another term for a qualitative code book?

8. In table 1, please include n's as well as %'s. I.e., report as "n (%)" and put the total n for each in the column for each participant type i.e., under service user sample put n = 30. The note under the table explaining why there are only percentages is not a convincing rationale. Anyone can do the math.

9. The supplementary materials are very fuzzy. Before publication, crisper graphics are recommended.

Reviewer 2

Name Agarwal, Anish

Affiliation University of Pennsylvania Health System, Department of Emergency Medicine

Date 06-Nov-2024

COI None

Interesting and appropriate approach.

Some general comments, the manuscript is quite long - even for a qualitative study. I think much of the introduction could be shortened and made more concise. A majority of the introduction can also be moved to methods if not removed entirely.

Please comment on why the market research company Leftfield was used. Were others considered? Could you recruit without a company?

Can you provide a kappa for coding?

Please speak to why just 8 HCPs

Nice presentation of themes, sub themes, and quotes

Discussion is nice, would benefit from interpreting these new findings within the context of what is known and perhaps pushing for potential future applications on how to best implement these practices for users and HCPs in the future.

VERSION 1 - AUTHOR RESPONSE

Reviewer 1

Dr. Bethany M Kwan, Department of Family Medicine, University of Colorado School of Medicine, Aurora, CO, USA.

Comments to the Author:
Overall summary and critique: This is a very well written and interesting paper on qualitative analysis of the experience of UK adults (both service users and health care practitioners) with the face to face Health Check and digital Health Check (an entirely digital, asynchronous version of risk assessment) implemented by the National Health Service. The analysis appears rigorously done and the conclusions are appropriate. I have a few minor critiques and suggestions:

1. **In the abstract's Objectives section, there is reference to the aim of the study including assessment of "barriers and facilitators to pathways". Then on page 6, it says the aims were to "identify barriers and facilitators to systems". In addition to this inconsistency, it's not clear which systems and pathways are being referred to. Please edit for consistency and clarity.**

The aim of the study was to identify barriers and facilitators to the face-to-face and digital Health Check pathways.

To clarify this, the Objectives section in the abstract has been amended to; 'This qualitative study aimed to understand service users' and Healthcare Professionals' (HCPs) experiences and opinions of F2F Health Checks and digital Health Checks (DHC), **identify barriers and facilitators of the face-to-face and digital Health Check pathways,** and recommend potential improvements.' (Page 2, Line 25-28)

To improve consistency the introduction section has been amended to 'This qualitative study aimed to understand service users' and Healthcare Professionals experiences and opinions of F2F Health Checks and DHCs, **identify barriers and facilitators of the F2F and DHC pathways**, and recommend potential improvements.' (Page 5, Line 114-116)

2. **In the abstract, please state how/by whom the participants were recruited.**

The following sentence has been added to the abstract; '**A purposive sample of 30 service users and 8 HCPs were recruited by an external market service company, in the London Borough of Southwark**.' (Page 2, Line 30-31)

3. **In the introduction, there is a likely typo - extra "ed" after "transferred" on page 4.**

The authors have amended the typo. (Page 4, Line 76).

4. **In the study design, please explain what made this a purposive sample.**

To clarify how this is a purposive sample the following sentence has been added to the study design section in the methods; 'A qualitative design was adopted for this research using one-to-one semi-structured interviews with a purposive sample of service users (**individuals who**

had been invited to either F2F or DHC Health Check) and **relevant Healthcare Professionals (HCPs) from the target area identified by GP practice managers**.' (Page 5-6, Line 120-124)

The authors note that the sampling is described in detail in the section on participant recruitment in the methods (Page 6-7, Line 148-163).

5. **On page 7 under participant recruitment, there is mention that invitations to complete the Health Check were sent to 9000 people. This is a bigger ask, but I would like to suggest including an analysis of who received the invitations to complete the Health Checks and who actually attended an appointment, including demographics. Especially given the limitations of the qualitative data, the reach of the service (especially who did not participate and whether the sample interviewed was representative of those who used the services) is important to understand.**

The authors acknowledge that this is a key piece of information that is important for context within this study. Since this paper was put into review another manuscript detailing the full service evaluation of the uptake and effectiveness of the digital health check pathway has been published (Salway et al., 2024). This paper provides a detailed analysis of who received the invitations, who attended and the demographics of these individuals. The authors have included a reference to this paper in the introduction (Page 5, Line 112) and methods sections (Page 6, Line 147).

A summary of the demographic information of those who attended the Health Checks has also been added to the Participant Recruitment section of the methods:

'Overall, 20% of patients completed a Health Check. Of those assigned to DHC, 21% completed the DHC Health Check, and a further 3% chose to complete a F2F Health Check, compared to 11% of those assigned to F2F who completed a F2F Health Check. Those who completed any type of Health Check included higher proportions of women, those with a family history of CVD and those from less deprived areas. Those who completed a DHC compared to a F2F health check included more men, those from white ethnicity and those with low diabetes risk and fewer with overweight or obesity. A full breakdown of demographic information of those involved in the service evaluation is reported elsewhere[15].' (Page 6, Line 139-147)

6. **Please add a section on Materials in the methods section and explain the development and content of the interview guides used for both the patient and HCP interviews. Please also specify in the study design section the intended number of interviews for both types of participants.**

A section on Materials has been included in the methods (Page 7-8, Line 174-186). Information about the interview schedules has been moved from the Procedure section and put into the Materials section.

'Materials

Interview schedules for service user and HCP interviews were co-developed by the whole project team, with input from the Public and Patient Involvement group (see 'Patient and Public Involvement'). Interview schedules were created based on the intervention logic model and study research aims. Briefly, service user interviews sought to understand why service users chose either a F2F Health Check or Digital Health Check or neither, their experience of the service and in what ways the service could be

improved. Interviews with HCPs covered their experience of providing the combined Health Check service, including any impact on workload for them and their colleagues, any concerns or perceived benefits of the service, and any suggestions for improvements. The research team revised the schedules based on the progress of early interviews (e.g., including more information about the DHC for HCPs who were not aware of them). The final interview schedules for both service users and HCPs are presented in the Supplementary Materials section'

The following sentence has been included in the Participant Recruitment section **'The study aimed to recruit 30 service users and 10 HCPs'.** (Page 7, Line 163)

7. **Please clarify, what are "draft thematic frameworks"? Is this another term for a qualitative code book?**

A draft thematic framework is another term for a qualitative code book. It is referred to as an analytical framework in the Framework method. To improve clarity the authors have changed 'thematic framework' to 'analytical framework' throughout. Additionally, the following explanation will be included in the analysis section; '**In the Framework method a qualitative code book is referred to as an analytical framework. This is created when the researchers have coded the first few transcripts independently and then meet to compare labels and agree on a set of codes to apply to all subsequent transcripts. These codes can be grouped into categories and are clearly defined[19]'.** (Page 8, Line 199-203).

8. **In table 1, please include n's as well as %'s. I.e., report as "n (%)" and put the total n for each in the column for each participant type i.e., under service user sample put n = 30. The note under the table explaining why there are only percentages is not a convincing rationale. Anyone can do the math**

The authors have replaced the percentages with n in the table for clarity. To protect anonymity, 'non-binary' has been removed from the table and an explanatory sentence has been added as a footnote to Gender*; '*Excluding other, refused or not reported'. (Page 9, Line 220-221).

9. **The supplementary materials are very fuzzy. Before publication, crisper graphics are recommended.**

Better quality graphics have been included.

Reviewer: 2

Dr. Anish Agarwal, University of Pennsylvania Health System
Comments to the Author:
Interesting and appropriate approach.
Some general comments, the manuscript is quite long - even for a qualitative study. I think much of the introduction could be shortened and made more concise. A majority of the introduction can also be moved to methods if not removed entirely.

The authors have edited and synthesized the Introduction section from 1085 words to 672 words.

Please comment on why the market research company Leftfield was used. Were others considered? Could you recruit without a company?

The market research company Leftfield was used on the advice of Southwark Local Council as their experience on other projects was that external companies led to greater recruitment. No other companies were considered.

Can you provide a kappa for coding?

A kappa for coding was not used for this study. The research team followed the Framework Method for analyses as outlined in Gale et al., 2013. This method outlines a step-by-step procedure for analyses; Stage 3: Coding: at least two researchers independently code the first few manuscripts. Then Stage 4: Developing a working analytical framework: after coding the first few transcripts, researchers meet up to compare labels and agree on a set of codes to apply to all subsequent transcripts.

To improve clarity on this procedure the following sentence has been added to the analysis section '**In the Framework method a qualitative code book is referred to as an analytical framework. This is created when the researchers have coded the first few transcripts independently and then meet to compare labels and agree on a set of codes to apply to all subsequent transcripts. These codes can be grouped into categories and are clearly defined**[19]' (Page 8, Line 199-203).

Please speak to why just 8 HCPs

The study intended to recruit 10 HCPs but were unable to do so. This is acknowledged in the limitations section. (Page 19, Line 503).

Nice presentation of themes, sub themes, and quotes
Discussion is nice, would benefit from interpreting these new findings within the context of what is known and perhaps pushing for potential future applications on how to best implement these practices for users and HCPs in the future.

Additional studies have been included in the discussion to interpret the findings within the context of what is already known. The following sentences and evidence has been added into the discussion section:

'Motivation and attitudes has been highlighted in previous studies as an important factor for benefiting digital services[25-27].' (Page 17, Line 451-452).

'A qualitative study with GPs found one of the key concerns with using digital, artificial intelligence systems with patients was losing the doctor-patient relationship[28].' (Page 17, Line 463-465).

'Previous research on digital healthcare, particularly for mental health services where this doctor-patient relationships are vital, found this lack of interaction with HCPs in the digital sphere a big challenge[35-37].' (Page 17-18, Line 469-471).

The following sentence was amended in the conclusion section to highlight how to better implement these practices for HCPs and users in the future.

'To improve the implementation of the DHC in the future, the following recommendations have been suggested based on the study findings: communicate problematic results and advice in person, provide an opportunity for discussion; and raise awareness among HCPs of the DHC as a complementary service to the F2F Health Checks and its potential to address the challenges

experienced by GP clinics. These recommendations may increase acceptability of the DHC overall and facilitate its implementation in the healthcare system.' (Page 19, Line 515-521).

VERSION 2 - REVIEW

Reviewer **1**

Name **Kwan, Bethany M**

Affiliation **Department of Family Medicine, University of Colorado School of Medicine, Aurora, CO, USA.**

Date **30-Jan-2025**

COI

All of my previous comments and concerns have been addressed well. This is an excellent paper.
